# (*R*,*E*)-3-(4-Chloro­phen­yl)-1-phenyl­allyl 4-nitro­benzoate

**DOI:** 10.1107/S1600536812032813

**Published:** 2012-07-25

**Authors:** Konstantin Troshin, Peter Mayer, Herbert Mayr

**Affiliations:** a Ludwig-Maximilians-Universität, Department, Butenandtstrasse 5–13, 81377 München, Germany

## Abstract

The title compound, C_22_H_16_ClNO_4_, adopts a conformation in which the phenyl ring plane forms similar dihedral angles with the nitro­benzoate C_6_ ring [76.97 (8)°] and the chloro­phenyl group [76.95 (8)°]; the dihedral angle between the chloro­phenyl and nitro­benzoate rings is 66.43 (8)°. In the crystal, π–π stacking is observed between the latter two planes, with a dihedral angle of 1.79 (8)° and a centroid–centroid distance of 3.735 (1) Å. In addition, mol­ecules are linked along [100] by weak C—H⋯O contacts.

## Related literature

For background to the stereochemistry of allylic rearrangements, see: Hughes (1941 ▶); Raber *et al.* (1974 ▶); Goering *et al.* (1971 ▶). For details of the synthesis, see: Troshin *et al.* (2011 ▶); Gao *et al.* (1987 ▶); Roos & Donovan (1996 ▶). For related structures, see: Cao *et al.* (2011 ▶); Wang *et al.* (2009 ▶). 
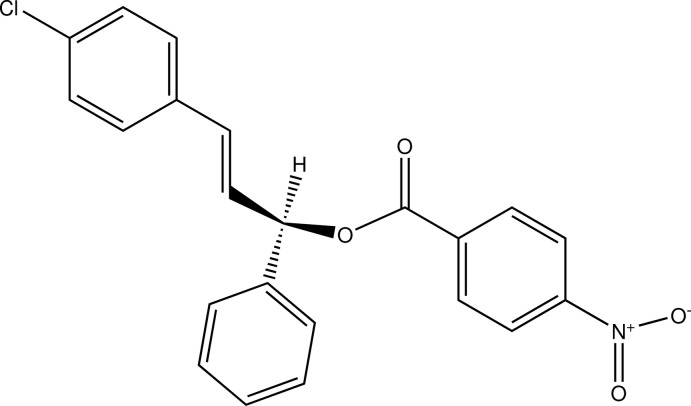


## Experimental

### 

#### Crystal data

C_22_H_16_ClNO_4_
*M* *_r_* = 393.82
Orthorhombic, 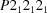

*a* = 8.3817 (1) Å
*b* = 9.9238 (2) Å
*c* = 22.8090 (4) Å
*V* = 1897.21 (6) Å^3^
*Z* = 4
Mo *K*α radiation
μ = 0.23 mm^−1^
*T* = 173 K
0.28 × 0.15 × 0.13 mm

#### Data collection

Nonius KappaCCD diffractometer
12403 measured reflections
4331 independent reflections
3847 reflections with *I* > 2σ(*I*)
*R*_int_ = 0.030

#### Refinement

*R*[*F* ^2^ > 2σ(*F* ^2^)] = 0.033
*wR*(*F* ^2^) = 0.083
*S* = 1.03
4331 reflections
253 parameters
H-atom parameters constrained
Δρ_max_ = 0.17 e Å^−3^
Δρ_min_ = −0.19 e Å^−3^
Absolute structure: Flack (1983) ▶, 1854 Friedel pairs
Flack parameter: 0.01 (5)

### 

Data collection: *COLLECT* (Hooft, 1998 ▶); cell refinement: *SCALEPACK* (Otwinowski & Minor, 1997 ▶); data reduction: *DENZO* (Otwinowski & Minor, 1997 ▶) and *SCALEPACK*; program(s) used to solve structure: *SIR97* (Altomare *et al.*, 1999 ▶); program(s) used to refine structure: *SHELXL97* (Sheldrick, 2008 ▶); molecular graphics: *ORTEP-3* (Farrugia, 1997 ▶); software used to prepare material for publication: *PLATON* (Spek, 2009 ▶).

## Supplementary Material

Crystal structure: contains datablock(s) I, global. DOI: 10.1107/S1600536812032813/gg2092sup1.cif

Structure factors: contains datablock(s) I. DOI: 10.1107/S1600536812032813/gg2092Isup2.hkl

Supplementary material file. DOI: 10.1107/S1600536812032813/gg2092Isup3.cml

Additional supplementary materials:  crystallographic information; 3D view; checkCIF report

## Figures and Tables

**Table 1:** Hydrogen-bond geometry (Å, °)

*D*—H⋯*A*	*D*—H	H⋯*A*	*D*⋯*A*	*D*—H⋯*A*
C2—H2⋯O4^i^	0.95	2.59	3.529 (2)	168
C12—H12⋯*Cg* ^ii^	0.95	2.92	3.8072 (19)	157

Symmetry codes: (i) 
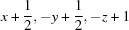
; (ii) 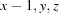.

**Fig. 1.**
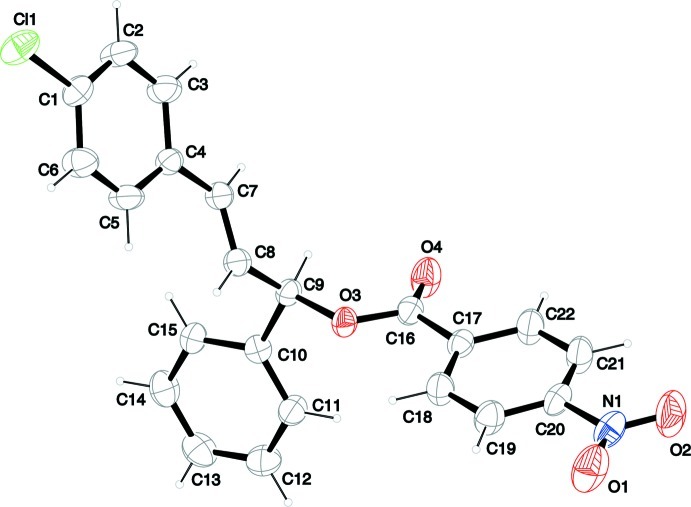
The molecular structure of the title compound, with atom labels and anisotropic displacement ellipsoids (drawn at 50% probability level) for non-H atoms.

**Fig. 2.**
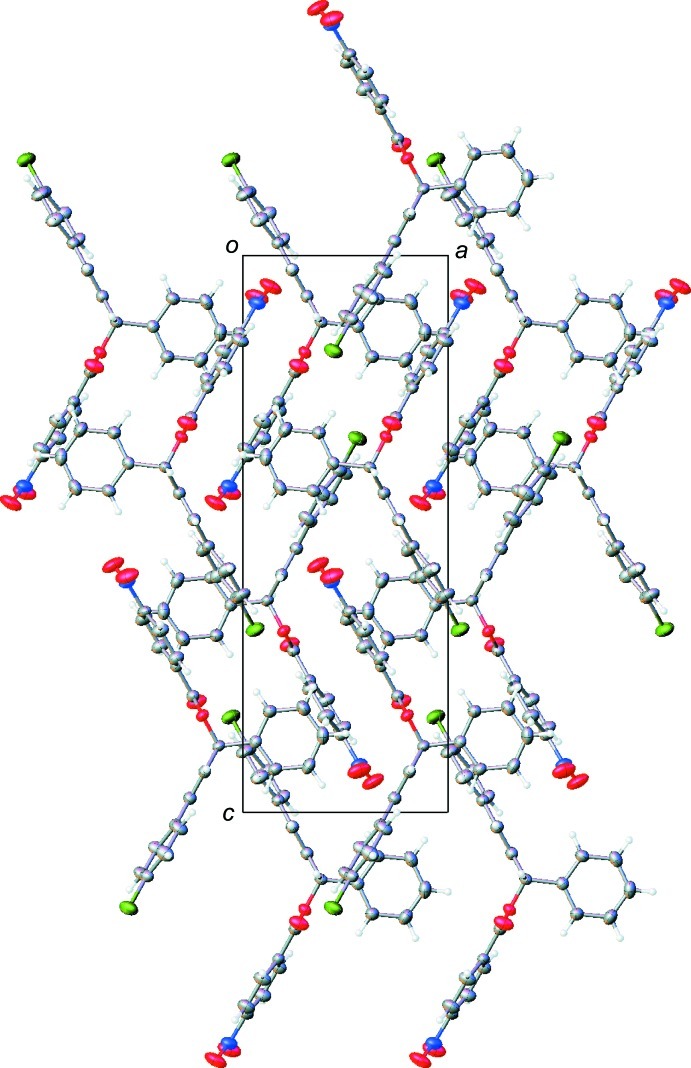
The packing of the title compound viewed along [0 - 1 0].

**Fig. 3.**
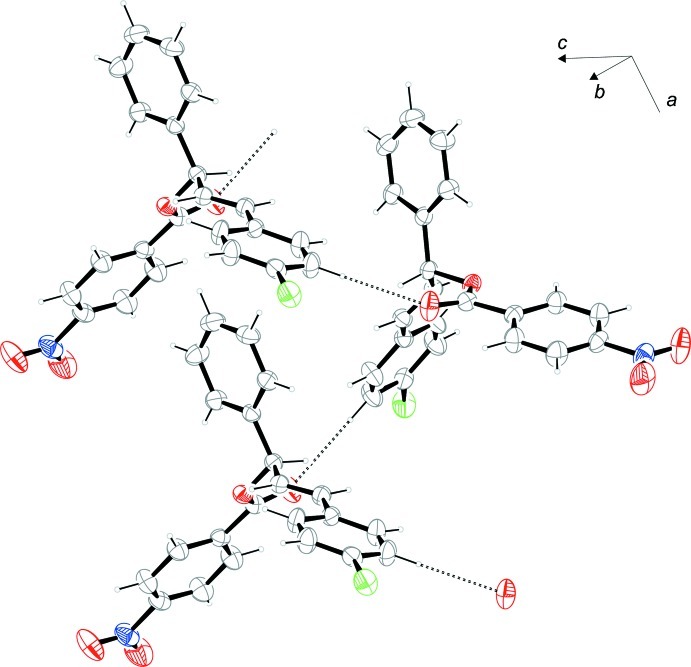
Weak C–H···O contacts linking the molecules along [100].

## supplementary crystallographic information

### Comment

Allylic substances have been used since 1940s to get an insight into the detailed mechanism of S_N_1 reactions due to the possibility of allylic rearrangement which gives additional analytical probe not available for other systems [Hughes (1941); Raber et al. (1974)]. Pioneering research of Goering et al. (1971) on the solvolyses of optically active allyl derivatives provided lots of information about ion-pairing in S_N_1 solvolyses (Goering et al., 1971), however, due to the lack of analytical methods available during Goering's time, many questions about ion pair dynamics and stereochemistry of allylic rearrangements remained open. As modern analytical techniques such as chiral HPLC and laser-flash photolysis give unprecedented insights in the reaction kinetics, we decided to reconsider the problem of stereochemistry of allylic rearrangements. The title compound (R,E)-3-(4-chlorophenyl)-1-phenylallyl 4-nitrobenzoate was chosen as a model compound for this research, as all eight compounds which can be present in the system during its solvolysis in aqueous solvents (i.e. (R) and (S) isomers of title compound, (<I>E)-1-(4-chlorophenyl)-3-phenylallyl 4-nitrobenzoate, 3-(4-chlorophenyl)-1-phenylprop-2-en-1-ol, and 1-(4-chlorophenyl)-3-phenylprop-2-en-1-ol)) can be resolved using chiral HPLC. X-ray diffraction analysis was used to confirm the absolute configuration of the title compound (I).

The asymmetric unit contains one molecule of the title compound (I) which is shown in Figure 1. The plane defined by C4, C7, C8 and C9 (contains the allyl group) is not exactly coplanar with the adjacent chlorophenyl moiety (dihedral angle 9.71 (17)°). A deviation of coplanarity of a similar magnitude (6.5 (3)°) is observed in a related structure with a phenyl group as adjacent moiety [Cao et al. 2011)], while a less deviation (dihedral angle 1.7 (6)°) is observed in a related structure with a p-toluyl group as adjacent moiety [Wang et al. (2009)]. A slight deviation from coplanarity is observed in the nitrobenzoate group as well. The plane of the nitro group is almost coplanar with the phenyl ring enclosing a dihedral angle of 0.9 (2)°. However, the plane of the CO_2_ group forms a dihedral angle of 10.16 (19)° with the phenyl ring.

The packing of (I) is shown in Figure 2. π-stacking is established between the chlorophenyl and nitrobenzoate moieties. These planes are arranged almost parallel to the viewing direction of Figure 2. A C-H···π contact is noted between C12-H12 and the nitrobenzoate moiety (distance C12···Cg 3.807 (2) Å). A weak C–H···O hydrogen bond is formed between C2 and O4 with a donor-acceptor distance of 3.529 (2) Å linking the molecules along [100] (see Fig. 3).

### Experimental

A four step synthesis was used to obtain the title compound (I).

1) (*E*)-3-(4-Chlorophenyl)-1-phenyl-prop-2-enone (18.1 g, 74.7 mmol, 87.2%) was synthesized from acetophenone (10.3 g, 85.7 mmol) and 4-chlorobenzaldehyde (12.0 g, 85.7 mmol) using an aldol condensation [Troshin *et al.* (2011)].

2) (*E*)-3-(4-Chlorophenyl)-1-phenyl-prop-2-enone (16.6 g, 68.4 mmol) was reduced with sodium borohydride (*ca* 5.0 g, *ca* 140 mmol) yielding the racemic (*E*)-3-(4-Chlorophenyl)-1-phenyl-prop-2-en-1-ol (15.7 g, 64.2 mmol, 94%) [Troshin *et al.* (2011)].

3) The (*R*) isomer of (*E*)-3-(4-Chlorophenyl)-1-phenyl-prop-2-en-1-ol was obtained by a Sharpless epoxidation of racemic (*E*)-3-(4-Chlorophenyl)-1-phenyl-prop-2-en-1-ol (15.5 g, 63.3 mmol) with D(-)-DIPT (1.78 g, 7.60 mmol), titanium(IV)isopropoxide (1.81 g, 6.33 mmol), and *^t^*BuOOH (8.4 ml of the 4.9 *M* CH_2_Cl_2_ solution [Gao *et al.* (1987)], 41.1 mmol) [Roos *et al.* (1996)]. The crude mixture was separated from the solvents and dissolved in ethanol followed by addition of piperidine (6.3 ml, 5.4 g, 64 mmol) and then refluxed for 10 h. The resulting solution was washed with 0.2 *M* aq HCl and water and then purified using column chromatography (silica gel, isohexane/diethyl ether) yielding *R,E*)-3-(4-Chlorophenyl)-1-phenyl-prop-2-en-1-ol (4.65 g, 19.0 mmol, 30%).

4) *R,E*)-3-(4-Chlorophenyl)-1-phenyl-prop-2-en-1-ol (3.00 g, 12.3 mmol) was dissolved in dichloromethane followed by addition of triethylamine (2.7 ml, 2.0 g, 19 mmol), DMAP (195 mg, 1.60 mmol), and 4-nitrobenzoyl chloride (2.96 g, 15.9 mmol) and the resulting solution was stirred for 30 min. The reaction mixture was washed with 0.2 *M* aq HCl and water, separated from the solvent, and the crude product was recrystallized twice from dichloromethane/pentane yielding (I) (1.90 g, 4.82 mmol, 39.4%) of > 99% ee (HPLC).

### Refinement

C-bound H atoms were positioned geometrically in ideal distances (0.95 Å for aromatic H and 1.00 Å for aliphatic H) and treated as riding on their parent atoms [*U*_iso_(H) = 1.2*U*_eq_(C)].

### Crystal data

**Table:**

C_22_H_16_ClNO_4_	*F*(000) = 816
*M**_r_* = 393.82	*D*_x_ = 1.38 Mg m^−^^3^
Orthorhombic, *P*2_1_2_1_2_1_	Mo *K*α radiation, λ = 0.71073 Å
Hall symbol: P 2ac 2ab	Cell parameters from 6811 reflections
*a* = 8.3817 (1) Å	θ = 3.1–27.5°
*b* = 9.9238 (2) Å	µ = 0.23 mm^−^^1^
*c* = 22.8090 (4) Å	*T* = 173 K
*V* = 1897.21 (6) Å^3^	Block, colourless
*Z* = 4	0.28 × 0.15 × 0.13 mm

### Data collection

**Table:**

Nonius KappaCCD diffractometer	3847 reflections with *I* > 2σ(*I*)
Radiation source: rotating anode	*R*_int_ = 0.030
MONTEL, graded multilayered X-ray optics monochromator	θ_max_ = 27.5°, θ_min_ = 3.2°
Detector resolution: 9 pixels mm^-1^	*h* = −10→10
CCD; rotation images; thick slices scans	*k* = −12→11
12403 measured reflections	*l* = −29→28
4331 independent reflections	

### Refinement

**Table:**

Refinement on *F*^2^	Secondary atom site location: difference Fourier map
Least-squares matrix: full	Hydrogen site location: inferred from neighbouring sites
*R*[*F*^2^ > 2σ(*F*^2^)] = 0.033	H-atom parameters constrained
*wR*(*F*^2^) = 0.083	*w* = 1/[σ^2^(*F*_o_^2^) + (0.039*P*)^2^ + 0.3359*P*] where *P* = (*F*_o_^2^ + 2*F*_c_^2^)/3
*S* = 1.03	(Δ/σ)_max_ = 0.001
4331 reflections	Δρ_max_ = 0.17 e Å^−^^3^
253 parameters	Δρ_min_ = −0.19 e Å^−^^3^
0 restraints	Absolute structure: Flack (1983), 1854 Friedel pairs
Primary atom site location: structure-invariant direct methods	Flack parameter: 0.01 (5)

### Special details

**Table:**

Geometry. All e.s.d.'s (except the e.s.d. in the dihedral angle between two l.s. planes) are estimated using the full covariance matrix. The cell e.s.d.'s are taken into account individually in the estimation of e.s.d.'s in distances, angles and torsion angles; correlations between e.s.d.'s in cell parameters are only used when they are defined by crystal symmetry. An approximate (isotropic) treatment of cell e.s.d.'s is used for estimating e.s.d.'s involving l.s. planes.
Refinement. Refinement of *F*^2^ against ALL reflections. The weighted *R*-factor *wR* and goodness of fit *S* are based on *F*^2^, conventional *R*-factors *R* are based on *F*, with *F* set to zero for negative *F*^2^. The threshold expression of *F*^2^ > 2*σ*(*F*^2^) is used only for calculating *R*-factors(gt) *etc*. and is not relevant to the choice of reflections for refinement. *R*-factors based on *F*^2^ are statistically about twice as large as those based on *F*, and *R*-factors based on all data will be even larger.

### Fractional atomic coordinates and isotropic or equivalent isotropic displacement parameters (Å^2^)

**Table:**

	*x*	*y*	*z*	*U*_iso_*/*U*_eq_	
Cl1	1.05905 (6)	−0.23586 (5)	0.67154 (2)	0.04970 (13)	
O1	1.0670 (2)	−0.12603 (15)	0.07501 (6)	0.0654 (4)	
O2	1.1347 (2)	0.07816 (17)	0.05567 (6)	0.0674 (4)	
O3	0.69865 (12)	0.07772 (10)	0.32577 (5)	0.0306 (2)	
O4	0.72705 (18)	0.29656 (12)	0.30153 (5)	0.0476 (3)	
N1	1.0677 (2)	−0.00643 (17)	0.08602 (6)	0.0456 (4)	
C1	0.9715 (2)	−0.14740 (16)	0.61393 (7)	0.0348 (4)	
C2	0.9926 (2)	−0.01089 (18)	0.61008 (8)	0.0433 (4)	
H2	1.0551	0.0358	0.6383	0.052*	
C3	0.9209 (2)	0.05778 (18)	0.56419 (7)	0.0423 (4)	
H3	0.9343	0.1526	0.5614	0.051*	
C4	0.82999 (18)	−0.00806 (16)	0.52214 (7)	0.0308 (3)	
C5	0.8104 (2)	−0.14624 (17)	0.52765 (8)	0.0403 (4)	
H5	0.7480	−0.1936	0.4996	0.048*	
C6	0.8806 (2)	−0.21628 (17)	0.57359 (8)	0.0441 (4)	
H6	0.8662	−0.3109	0.5771	0.053*	
C7	0.75987 (18)	0.07074 (16)	0.47422 (7)	0.0317 (3)	
H7	0.7667	0.1660	0.4776	0.038*	
C8	0.68791 (18)	0.02343 (16)	0.42667 (7)	0.0304 (3)	
H8	0.6799	−0.0714	0.4218	0.036*	
C9	0.61891 (17)	0.11246 (16)	0.38042 (6)	0.0289 (3)	
H9	0.6421	0.2087	0.3903	0.035*	
C10	0.43980 (17)	0.09433 (14)	0.37273 (6)	0.0284 (3)	
C11	0.36984 (18)	0.08652 (17)	0.31807 (7)	0.0357 (4)	
H11	0.4345	0.0910	0.2839	0.043*	
C12	0.2058 (2)	0.07210 (19)	0.31259 (8)	0.0441 (4)	
H12	0.1587	0.0677	0.2748	0.053*	
C13	0.11123 (19)	0.06417 (19)	0.36165 (9)	0.0438 (4)	
H13	−0.0009	0.0534	0.3578	0.053*	
C14	0.1797 (2)	0.07192 (18)	0.41646 (8)	0.0409 (4)	
H14	0.1145	0.0669	0.4505	0.049*	
C15	0.34379 (19)	0.08698 (16)	0.42224 (7)	0.0335 (3)	
H15	0.3904	0.0923	0.4601	0.040*	
C16	0.74556 (18)	0.17917 (16)	0.29088 (7)	0.0308 (3)	
C17	0.82662 (18)	0.12685 (16)	0.23721 (6)	0.0294 (3)	
C18	0.8264 (2)	−0.00856 (17)	0.22281 (7)	0.0377 (4)	
H18	0.7725	−0.0714	0.2472	0.045*	
C19	0.9049 (2)	−0.05269 (17)	0.17272 (8)	0.0417 (4)	
H19	0.9046	−0.1453	0.1621	0.050*	
C20	0.98271 (19)	0.04050 (17)	0.13903 (7)	0.0354 (4)	
C21	0.9847 (2)	0.17555 (18)	0.15197 (8)	0.0440 (4)	
H21	1.0391	0.2378	0.1275	0.053*	
C22	0.9051 (2)	0.21839 (17)	0.20181 (7)	0.0412 (4)	
H22	0.9045	0.3114	0.2117	0.049*	

### Atomic displacement parameters (Å^2^)

**Table:**

	*U*^11^	*U*^22^	*U*^33^	*U*^12^	*U*^13^	*U*^23^
Cl1	0.0626 (3)	0.0493 (2)	0.0372 (2)	0.0150 (2)	−0.0132 (2)	−0.00003 (18)
O1	0.0983 (12)	0.0535 (8)	0.0444 (8)	0.0182 (9)	0.0219 (8)	−0.0061 (6)
O2	0.0855 (11)	0.0720 (10)	0.0446 (8)	−0.0062 (9)	0.0325 (7)	−0.0015 (7)
O3	0.0303 (5)	0.0345 (6)	0.0270 (5)	0.0005 (5)	0.0067 (4)	−0.0003 (5)
O4	0.0703 (9)	0.0339 (6)	0.0387 (7)	0.0041 (6)	0.0168 (6)	−0.0010 (5)
N1	0.0510 (9)	0.0568 (10)	0.0289 (7)	0.0100 (8)	0.0067 (7)	−0.0020 (7)
C1	0.0376 (9)	0.0380 (8)	0.0288 (8)	0.0088 (7)	−0.0014 (6)	−0.0007 (6)
C2	0.0531 (11)	0.0413 (9)	0.0356 (9)	−0.0003 (8)	−0.0154 (8)	−0.0076 (7)
C3	0.0562 (11)	0.0324 (8)	0.0384 (9)	−0.0044 (8)	−0.0105 (8)	−0.0031 (7)
C4	0.0298 (7)	0.0356 (8)	0.0269 (7)	−0.0018 (7)	0.0005 (6)	−0.0023 (6)
C5	0.0466 (10)	0.0359 (9)	0.0386 (9)	−0.0052 (8)	−0.0135 (8)	−0.0055 (7)
C6	0.0556 (11)	0.0310 (8)	0.0458 (10)	−0.0002 (8)	−0.0129 (8)	−0.0007 (7)
C7	0.0311 (7)	0.0333 (8)	0.0305 (7)	−0.0013 (7)	−0.0004 (6)	−0.0011 (6)
C8	0.0284 (7)	0.0324 (8)	0.0303 (8)	−0.0001 (6)	0.0011 (6)	−0.0013 (6)
C9	0.0289 (7)	0.0346 (8)	0.0231 (7)	−0.0001 (6)	0.0036 (5)	−0.0029 (6)
C10	0.0287 (7)	0.0257 (7)	0.0309 (7)	0.0023 (6)	0.0020 (6)	−0.0005 (5)
C11	0.0338 (8)	0.0436 (9)	0.0297 (8)	0.0014 (7)	0.0005 (6)	0.0013 (7)
C12	0.0356 (9)	0.0536 (11)	0.0430 (10)	0.0005 (9)	−0.0097 (7)	0.0026 (8)
C13	0.0278 (8)	0.0453 (10)	0.0583 (12)	0.0016 (7)	−0.0014 (8)	0.0037 (9)
C14	0.0332 (8)	0.0429 (9)	0.0466 (10)	0.0031 (8)	0.0119 (7)	0.0015 (8)
C15	0.0356 (8)	0.0369 (8)	0.0281 (8)	0.0008 (7)	0.0047 (6)	0.0007 (6)
C16	0.0301 (8)	0.0358 (8)	0.0265 (7)	0.0014 (6)	0.0001 (6)	0.0029 (6)
C17	0.0269 (7)	0.0356 (8)	0.0258 (7)	0.0036 (6)	−0.0006 (6)	0.0019 (6)
C18	0.0424 (9)	0.0363 (8)	0.0344 (9)	−0.0009 (8)	0.0073 (7)	0.0014 (7)
C19	0.0536 (10)	0.0361 (8)	0.0352 (9)	0.0026 (8)	0.0067 (8)	−0.0027 (7)
C20	0.0354 (8)	0.0467 (9)	0.0239 (7)	0.0081 (7)	0.0038 (6)	0.0011 (6)
C21	0.0538 (11)	0.0435 (9)	0.0348 (9)	−0.0023 (8)	0.0129 (8)	0.0045 (7)
C22	0.0542 (10)	0.0350 (9)	0.0343 (9)	−0.0004 (8)	0.0107 (8)	0.0019 (7)

### Geometric parameters (Å, º)

**Table:**

Cl1—C1	1.7424 (16)	C9—H9	1.0000
O1—N1	1.213 (2)	C10—C11	1.380 (2)
O2—N1	1.224 (2)	C10—C15	1.389 (2)
O3—C16	1.3422 (18)	C11—C12	1.388 (2)
O3—C9	1.4557 (17)	C11—H11	0.9500
O4—C16	1.200 (2)	C12—C13	1.373 (3)
N1—C20	1.479 (2)	C12—H12	0.9500
C1—C2	1.369 (2)	C13—C14	1.378 (3)
C1—C6	1.376 (2)	C13—H13	0.9500
C2—C3	1.386 (2)	C14—C15	1.389 (2)
C2—H2	0.9500	C14—H14	0.9500
C3—C4	1.388 (2)	C15—H15	0.9500
C3—H3	0.9500	C16—C17	1.493 (2)
C4—C5	1.387 (2)	C17—C22	1.382 (2)
C4—C7	1.467 (2)	C17—C18	1.383 (2)
C5—C6	1.389 (2)	C18—C19	1.389 (2)
C5—H5	0.9500	C18—H18	0.9500
C6—H6	0.9500	C19—C20	1.368 (2)
C7—C8	1.327 (2)	C19—H19	0.9500
C7—H7	0.9500	C20—C21	1.372 (2)
C8—C9	1.493 (2)	C21—C22	1.385 (2)
C8—H8	0.9500	C21—H21	0.9500
C9—C10	1.522 (2)	C22—H22	0.9500
			
C16—O3—C9	117.68 (12)	C10—C11—C12	120.54 (15)
O1—N1—O2	123.77 (16)	C10—C11—H11	119.7
O1—N1—C20	118.37 (15)	C12—C11—H11	119.7
O2—N1—C20	117.86 (16)	C13—C12—C11	120.26 (16)
C2—C1—C6	121.35 (16)	C13—C12—H12	119.9
C2—C1—Cl1	119.49 (13)	C11—C12—H12	119.9
C6—C1—Cl1	119.15 (13)	C12—C13—C14	119.71 (15)
C1—C2—C3	118.60 (16)	C12—C13—H13	120.1
C1—C2—H2	120.7	C14—C13—H13	120.1
C3—C2—H2	120.7	C13—C14—C15	120.30 (16)
C2—C3—C4	121.90 (16)	C13—C14—H14	119.8
C2—C3—H3	119.1	C15—C14—H14	119.8
C4—C3—H3	119.1	C10—C15—C14	120.13 (15)
C5—C4—C3	117.89 (15)	C10—C15—H15	119.9
C5—C4—C7	123.18 (15)	C14—C15—H15	119.9
C3—C4—C7	118.93 (14)	O4—C16—O3	124.77 (14)
C4—C5—C6	120.87 (15)	O4—C16—C17	124.22 (14)
C4—C5—H5	119.6	O3—C16—C17	111.01 (13)
C6—C5—H5	119.6	C22—C17—C18	120.02 (15)
C1—C6—C5	119.38 (16)	C22—C17—C16	117.84 (14)
C1—C6—H6	120.3	C18—C17—C16	122.14 (14)
C5—C6—H6	120.3	C17—C18—C19	120.07 (15)
C8—C7—C4	127.06 (15)	C17—C18—H18	120.0
C8—C7—H7	116.5	C19—C18—H18	120.0
C4—C7—H7	116.5	C20—C19—C18	118.33 (15)
C7—C8—C9	122.99 (15)	C20—C19—H19	120.8
C7—C8—H8	118.5	C18—C19—H19	120.8
C9—C8—H8	118.5	C19—C20—C21	123.05 (15)
O3—C9—C8	106.69 (12)	C19—C20—N1	118.43 (15)
O3—C9—C10	109.04 (12)	C21—C20—N1	118.53 (15)
C8—C9—C10	113.18 (13)	C20—C21—C22	118.06 (16)
O3—C9—H9	109.3	C20—C21—H21	121.0
C8—C9—H9	109.3	C22—C21—H21	121.0
C10—C9—H9	109.3	C17—C22—C21	120.47 (16)
C11—C10—C15	119.05 (13)	C17—C22—H22	119.8
C11—C10—C9	121.99 (13)	C21—C22—H22	119.8
C15—C10—C9	118.96 (13)		
			
C6—C1—C2—C3	−0.3 (3)	C12—C13—C14—C15	−0.3 (3)
Cl1—C1—C2—C3	−179.48 (14)	C11—C10—C15—C14	0.0 (2)
C1—C2—C3—C4	−0.5 (3)	C9—C10—C15—C14	179.30 (15)
C2—C3—C4—C5	0.9 (3)	C13—C14—C15—C10	0.0 (3)
C2—C3—C4—C7	−178.96 (17)	C9—O3—C16—O4	0.3 (2)
C3—C4—C5—C6	−0.5 (3)	C9—O3—C16—C17	179.34 (11)
C7—C4—C5—C6	179.38 (16)	O4—C16—C17—C22	9.7 (2)
C2—C1—C6—C5	0.7 (3)	O3—C16—C17—C22	−169.42 (14)
Cl1—C1—C6—C5	179.90 (14)	O4—C16—C17—C18	−171.07 (18)
C4—C5—C6—C1	−0.3 (3)	O3—C16—C17—C18	9.8 (2)
C5—C4—C7—C8	−9.2 (3)	C22—C17—C18—C19	−0.1 (3)
C3—C4—C7—C8	170.68 (15)	C16—C17—C18—C19	−179.32 (15)
C4—C7—C8—C9	179.48 (14)	C17—C18—C19—C20	0.7 (3)
C16—O3—C9—C8	−136.58 (13)	C18—C19—C20—C21	−1.0 (3)
C16—O3—C9—C10	100.86 (15)	C18—C19—C20—N1	179.41 (15)
C7—C8—C9—O3	121.40 (15)	O1—N1—C20—C19	−1.1 (3)
C7—C8—C9—C10	−118.67 (16)	O2—N1—C20—C19	178.82 (17)
O3—C9—C10—C11	−17.5 (2)	O1—N1—C20—C21	179.29 (18)
C8—C9—C10—C11	−136.06 (15)	O2—N1—C20—C21	−0.8 (3)
O3—C9—C10—C15	163.25 (13)	C19—C20—C21—C22	0.6 (3)
C8—C9—C10—C15	44.68 (19)	N1—C20—C21—C22	−179.82 (16)
C15—C10—C11—C12	0.3 (3)	C18—C17—C22—C21	−0.3 (3)
C9—C10—C11—C12	−178.98 (16)	C16—C17—C22—C21	178.93 (16)
C10—C11—C12—C13	−0.6 (3)	C20—C21—C22—C17	0.1 (3)
C11—C12—C13—C14	0.6 (3)		

### Hydrogen-bond geometry (Å, º)

**Table:**

*D*—H···*A*	*D*—H	H···*A*	*D*···*A*	*D*—H···*A*
C2—H2···O4^i^	0.95	2.59	3.529 (2)	168
C12—H12···*Cg*^ii^	0.95	2.92	3.8072 (19)	157

Symmetry codes: (i) *x*+1/2, −*y*+1/2, −*z*+1; (ii) *x*−1, *y*, *z*.
